# Probing ADAMTS13 Substrate Specificity using Phage Display

**DOI:** 10.1371/journal.pone.0122931

**Published:** 2015-04-07

**Authors:** Karl C. Desch, Colin Kretz, Andrew Yee, Robert Gildersleeve, Kristin Metzger, Nidhi Agrawal, Jane Cheng, David Ginsburg

**Affiliations:** 1 Department of Pediatrics, University of Michigan, Ann Arbor, Michigan, United States of America; 2 Life Sciences Institute, University of Michigan, Ann Arbor, Michigan, United States of America; 3 Howard Hughes Medical Institute, Ann Arbor, Michigan, United States of America; 4 Department of Internal Medicine and Human Genetics, University of Michigan, Ann Arbor, Michigan, United States of America; National Cerebral and Cardiovascular Center, JAPAN

## Abstract

Von Willebrand factor (VWF) is a large, multimeric protein that regulates hemostasis by tethering platelets to the subendothelial matrix at sites of vascular damage. The procoagulant activity of plasma VWF correlates with the length of VWF multimers, which is proteolytically controlled by the metalloprotease ADAMTS13. To probe ADAMTS13 substrate specificity, we created phage display libraries containing randomly mutated residues of a minimal ADAMTS13 substrate fragment of VWF, termed VWF73. The libraries were screened for phage particles displaying VWF73 mutant peptides that were resistant to proteolysis by ADAMTS13. These peptides exhibited the greatest mutation frequency near the ADAMTS13 scissile residues. Kinetic assays using mutant and wild-type substrates demonstrated excellent agreement between rates of cleavage for mutant phage particles and the corresponding mutant peptides. Cleavage resistance of selected mutations was tested *in vivo* using hydrodynamic injection of corresponding full-length expression plasmids into VWF-deficient mice. These studies confirmed the resistance to cleavage resulting from select amino acid substitutions and uncovered evidence of alternate cleavage sites and recognition by other proteases in the circulation of ADAMTS13 deficient mice. Taken together, these studies demonstrate the key role of specific amino acids residues including P3-P2’ and P11’, for substrate specificity and emphasize the importance in flowing blood of other ADAMTS13–VWF exosite interactions outside of VWF73.

## Introduction

von Willebrand factor (VWF) is a multimeric plasma glycoprotein that functions as a critical regulator of hemostasis, both as a carrier for coagulation factor VIII and as a molecular bridge between circulating blood platelets and sites of vascular injury. Synthesized exclusively in endothelial cells and megakaryocytes, VWF is stored in endothelial Weibel-Palade bodies and platelet alpha granules and secreted upon activation[1]. The initial secreted molecules include the most highly multimeric and procoagulant form of VWF, termed ultra-large VWF (UL-VWF)[2]. UL-VWF is rapidly cleaved upon secretion into the circulation by the metalloprotease ADAMTS13[3, 4]. ADAMTS13 deficiency results in an accumulation of UL-VWF and is associated with the development of thrombotic thrombocytopenic purpura (TTP), a life threatening thrombotic microangiopathy[5].

The only known substrate for ADAMTS13 is VWF[6]. ADAMTS13 is synthesized in multiple cells but appears to be predominantly secreted from hepatic stellate cells[7, 8]. In circulation, ADAMTS13 cleaves VWF at the Y1605/M1606 peptide bond in the VWF A2 domain[9]. *In vitro*, chaotropic denaturants or shear stress is required for the cleavage of VWF by ADAMTS13. However, in the circulation, many other factors help regulate ADAMTS13 cleavage activity, including hydrodynamic forces, VWF multimer length, VWF platelet interactions and VWF glycosylation patterns[10–13]. Taken together, these factors are thought to coordinate a conformational change in multimeric VWF that exposes the otherwise cryptic scissile bond to ADAMTS13. VWF73 is a fragment of the VWF A2 domain (D1596—R1668) that contains both the known cleavage site Y1605-M1606, as well as a number of other subsites that are important for recognition by ADAMTS13[14]. As a minimal substrate for ADAMTS13, VWF73 is efficiently cleaved without the need for denaturants or mechanical forces [15] but may be missing other subsites for ADAMTS13 that play functional roles *in vivo*[16–18].

In order to probe ADAMTS13 substrate specificity, we generated randomly mutated VWF73 substrate phage display libraries [19]. These libraries contained phage expressing mutagenized VWF73 peptides and epitope tags fused to the M13 phage coat protein PIII. The expression of the epitope tags facilitated the separation of cleaved from uncleaved phage after exposure to ADAMTS13. The library was screened to select for phage particles displaying VWF73 variants that were resistant to ADAMTS13 proteolysis and thus retained the epitope tags on the PIII fusion protein. DNA sequencing of recovered enzyme-resistant phage demonstrated an increased frequency of mutations between the P3 and P11’ positions in VWF73, suggesting that these residues are critical for cleavage of the Y1605/M1606 scissile bond. These findings were confirmed in kinetic assays of mutant phage and of mutant VWF73 peptides. We also report the effects of these mutations on multimeric VWF in the murine circulation and demonstrate that select mutations confer ADAMTS13 resistance while other mutations promote ADAMTS13 independent proteolysis.

## Materials and Methods

### Phage Display Library Construction

The M13 filamentous phage display vector, fUSE55, was a gift from George P. Smith, University of Missouri, Columbia, MO (GenBank Accession AF317217). fUSE55 phage with in frame cDNA insertions express a fusion protein as part of the wild-type PIII protein with a valency of 3–5 copies per phage particle. A library of mutant VWF73 sequences was constructed for expression in the fUSE55 phage display system. In PCR reactions with VWF cDNA,[20] primer P1 and P2, Table 1 amplified the D1596 to R1688 region of VWF and added NH_2_-terminal FLAG and T7 epitope tags. Mutagenic PCR was performed according to previously published methods [21]. Alternatively, to create a third library, mutagenic PCR was performed using the same primers and template but using the Mutazyme II (Agilent, Santa Clara, CA) kit, according to the manufacturer’s instructions. The mutated VWF73 PCR products were cloned into the BglI restriction site of fUSE55. Ligation products were electroporated into MC1061 cells [22]. The library depth was quantified by the number of colony forming units per mL (CFU) on NZY plates supplemented with 40 μg/mL tetracycline. Colonies were randomly selected to confirm VWF73 insertion by DNA sequencing using primer P3 and P4, Table 1.

**Table 1:** Primer List.

Primer	Sequence 5’– 3’
P1	ATGTCGGCCGACGTGGCCGACTACAAGGACGATGACGATAAAGGAATGGCATCAATGACAGGAGGACAACAAATGGACCGGGAGCAGGCGCCCAAC
P2	GATGGCCCCAGAGGCCATCCTCTGCAGCACCAGGTCAGGA
P3	CACCTCGAAAGCAAGCTGATAAACCG
P4	CGCCTGTAGCATTCCACAGACAGCCC
P5	GGGCGATGGTTGTTGTCATTGTCGG
P6	TTTCAGGGATAGCAAGCCCAATAGG
P7	CCCAGCCAAGGGGACTGGGTAGAGGCACCTAAC
P8	GTTAGGTGCCTCTACCCAGTCCCCTTGGCTGGG
P9	GGGTAGAGGCACCTAACCAGGTCTACATGGTCACGGGG
P10	CCCCGTGACCATGTAGACCTGGTTAGGTGCCTCTACCC
P11	GGGTAGAGGCACCTAACCGGGTCTACATGGTCACGG
P12	CCGTGACCATGTAGACCCGGTTAGGTGCCTCTACCC
P13	GGGTAGAGGCACCTAACCTGGTCGACATGGTCACGGGGAACC
P14	GGTTCCCCGTGACCATGTCGACCAGGTTAGGTGCCTCTACCC
P15	GGGTAGAGGCACCTAACCTGGTCAACATGGTCACGGGGAACC
P16	GGTTCCCCGTGACCATGTTGACCAGGTTAGGTGCCTCTACCC
P17	GGGTAGAGGCACCTAACCTGGTCTACACGGTCACGGGGAACC
P18	GGTTCCCCGTGACCGTGTAGACCAGGTTAGGTGCCTCTACCC
P19	GCCTCTGATGAGAACAAGAGGTTGCCTGGAGACATCC
P20	GGATGTCTCCAGGCAACCTCTTGTTCTCATCAGAGGC
P21	GGTAGAGGCACCTAACCGGGTCAACATGGTCACGGGGAACCCCG
P22	CGGGGTTCCCCGTGACCATGTTGACCCGGTTAGGTGCCTCTACC
P23	GGCACCTAACCTGGTCAACACGGTCACGGGGAACCCCGCCTCTGAT
P24	ATCAGAGGCGGGGTTCCCCGTGACCGTGTTGACCAGGTTAGGTGCC
P25	GGGTAGAGGCACCTAACCTGGTCTCCACGGTCACGGGGAACCCCG
P26	CGGGGTTCCCCGTGACCGTGGAGACCAGGTTAGGTGCCTCTACCC

### Phage Library Purification

Phage were prepared using a triple PEG/NaCl precipitation method[23]. Transformed MC1061 cells were cultured overnight in NZY containing 20 μg/ml tetracycline at 37°C. The conditioned medium was cleared by centrifugation, and the phage were precipitated from the supernatant with 0.15 volume of PEG/NaCl (16.7% m/v PEG 8000 + 3.3M NaCl) at 4°C for 10 hr. Precipitated phage were collected by centrifugation (8,000 x g, 1 hr) and resuspended in TBS (50 mM Tris-HCl, pH 7.5, 150 mM NaCl); insoluble material was cleared by centrifugation (1,000 x g, 15 min). Phage were precipitated twice more before storage in TBS at 4°C.

### ADAMTS13 enzyme preparation

Human full length ADAMTS13 cDNA[5] cloned into pcDNA3.1 (Invitrogen) was used to transfect HEK 293T cells grown to ~80% confluence using FuGene6 (Roche). Transfected cells were incubated with serum free DMEM (Invitrogen) for 48 hr. Conditioned media were clarified by centrifugation and concentrated ~20-fold using 50 kDa Amicon Ultra filters (Millipore, Billerica, MA). To confirm expression of recombinant ADAMTS13 (rADAMTS13), the concentrated conditioned media (CCM) was analyzed by western blot using a monoclonal anti-ADAMTS13 antibody. Prior to phage selection, rADAMTS13-containing CCM was dialyzed into ADAMTS13 reaction buffer A (50 mM Hepes pH 7.4, 150 mM NaCl, 5 mM CaCl_2_, 0.1 μM ZnCl_2_) in Slide-A-Lyzer dialysis cassettes (Thermo Scientific, Rockford, IL). Commercially prepared ADAMTS13 (R&D Systems, Minneapolis, MN) was used in enzyme kinetic experiments.

### Phage library Screening

Fresh phage were prepared for each screen by infection of F–pilus positive E.*coli* (K91Kan strain) [24, 25] with purified phage and expansion in an overnight culture of NZY with 20 μg/ml tetracycline and 100 μg/ml kanamycin followed by two rounds of PEG/NaCl precipitation as described above. Freshly purified VWF73-displaying phage were immunopreicpitated against FLAG in binding buffer (50 mM Tris-HCl, pH 7.4, 150 mM NaCl, 1% Tween 20, 5% BSA) at 4°C, overnight. Beads bearing mutant VWF73 phage were washed with ADAMTS13 reaction buffer A and incubated with dialyzed rADAMTS13 CCM at 37°C for 1 hr. Beads were collected by centrifugation, washed and mixed with additional rADAMTS13 for a total of 4 incubations. The titer of the bead supernatant decreased from ~9 x 10^3^ CFU/ml to ~0.08 x 10^3^ CFU/ml over 4 enzyme cycles. Next, phage remaining bound to anti-FLAG beads were recovered by infection with fresh K91 E.*coli* for 15 min and then plated on NZY agar plates containing 40 μg/ml tetracycline to isolate individual phage clones for further analysis.

### Secondary ELISA screening of phage clones

Individual phage colonies were purified by PEG/NaCl precipitation and added to anti-FLAG antibody-coated microtiter plates (Sigma, St. Louis, MO) in blocking buffer and incubated for 2 hr at 22°C. Each phage clone was plated in duplicate. Plates were washed three times before incubating with 150 μl of prepared rADAMTS13 in the presence or absence of 25 mM EDTA for 1 hr at 37°C.

To assay for phage released from the FLAG plate by rADAMTS13, a standard sandwich ELISA was performed using 100 μl of the cleavage reaction captured with anti-fd bacteriophage antibody (Sigma, B7786) coated on 96 well assay plates (Costar 3370, Tewksbury MA) and detected with anti-M13 Protein VIII-HRP antibody (GE Healthcare, 27-9421-01) and 1-Step Ultra TMB ELISA solution (Thermo, Rockford, IL). The ELISA signal was measured at **λ**_abs_ = 450 nm and read using a ThermoMax microplate reader (Molecular Devices, Sunnyvale, CA). The relative cleavage for each phage clone was estimated by the ratio of signals from reactions with rADAMTS13 to those with rADAMTS13 and 25 mM EDTA. Phage that demonstrated reduced ELISA signals compared to wild type controls were selected for sequencing. Primers P5 and P6 were used in PCR reactions with phage template from an aliquot of phage culture to amplify VWF73 template. Purified amplicons were submitted for sequencing with primers P3 and P4, Table 1, at the DNA Sequencing Core, (University of Michigan, Ann Arbor, MI). Sequencing results were analyzed using the Lasergene software suite (DNASTAR, Madison, WI).

### Substrate Phage Kinetics

The phage used in the kinetic analysis were prepared using the double precipitation method [23] as outlined above from a 1 L overnight culture grown in LB containing 30 μg/ml tetracycline in a Fernbach flask. Phage were resuspended in TBS and the final phage titer was determined using K91 *E*.*coli* and standard procedures [23]. Purified phage bearing mutant VWF73 were added to ADAMTS13 reaction buffer B (20 mM Tris-HCl, 150 mM NaCl, 4 mM CaCl_2_, 10 μM ZnCl_2_, 1% BSA, pH 7.4) at concentrations ranging from 0.5 pM to 10 pM (at least 17,000-fold < K_M_). Depending on the VWF73 mutant, either 10 nM or 100 nM ADAMTS13 (R&D Systems, Minneapolis, MN) was added to initiate the reaction, performed at 37°C. At time points between 0 and 600 min, 10 μl aliquots of the reaction were removed into 10 μl stop buffer to terminate the reaction (ADAMTS13 reaction buffer B, 50 mM EDTA).

In order to measure the extent of VWF73 proteolysis by ADAMTS13 at each reaction time point, we developed custom AlphaLISA assays (Perkin Elmer, Waltham, MA) [26]. These assays used acceptor and donor bead-linked antibodies against epitopes NH3-terminal (FLAG in phage and GST in peptides) and COOH terminal (P8 protein in phage and 6xHIS in peptides) to the VWF73 peptide sequence. In the AlphaLISA assay, signals are generated by the proximity of the donor beads to the acceptor beads, which occurs maximally in an uncleaved peptide bearing both NH3- terminal and COOH terminal epitope tags. ADAMTS13 cleavage of VWF73 results in the separation of the 2 beads resulting in a lower AlphaLISA signal. Thus, measurement of AlphaLISA signals at each reaction time point provides a quantitation of the extent of proteolysis as a function of time, which was used to calculate kinetic rate constants. This system proved to be sensitive and allowed for the relatively rapid assessment of multiple mutant phage or peptide substrates.

For this assay anti-FLAG antibody conjugated acceptor beads were used with biotinylated anti-fd bacteriophage antibody (B2661 Sigma), and streptavidin coated donor beads. Twenty-five μl of a solution containing 20 μg/ml acceptor beads and 4 nM anti-Fd-bacteriophage antibody was mixed with AlphaLISA buffer (25 mM HEPES, 1 mg/ml Dextran-500, 0.5% TritonX-100, 0.5% bovine serum albumin, 0.05% Proclin-300) in wells of a 96 half-well plate (Perkin Elmer). Next, 5 μl of stopped phage reaction time points was added to the wells, and incubated for 60 min at RT. Then, 20 μl of a solution containing 40 μg/ml donor beads in AlphaLISA buffer was added, followed by a 30 min incubation at 22°C. The plates were read on an EnSpire 2300 Multilabel Plate Reader (Perkin Elmer) using an **λ**_ex_ = 680 nm and and **λ**_em_ = 615 nm. The change in AlphaLISA signal from ADAMTS13 reaction time points was compared to a standard curve generated for each mutant to calculate the concentration of uncleaved phage at each time point. Initial rates of cleavage were determined according to:

$$V=\left({\left[S\right]}_{0}-{\left[S\right]}_{t}\right)/t$$ (1)

Where V is the rate of cleavage, S_0_ is the initial substrate concentration, S is the concentration of substrate, and t is time. Given that for all VWF73 variants, [S]<0.01 x K_M_ for WT VWF73 (approximately 1.7 μM)[17], data were fit to the equation for direct k_cat_/K_M_ determination:

$$V/\left[E\right]=\left(k_{cat}/K_{M}\right)\left(\left[S\right]\right)$$ (2)

Where V is the rate of substrate cleavage at concentration S, E is the concentration of ADAMTS13, and (k_cat_/K_M_) is the catalytic efficiency constant.

### Peptide purification

VWF73 mutants were cloned into the pGEX6P-1 vector (GE Healthcare Life Sciences, Piscataway, NJ) with a COOH-terminal 6X-His tag and an NH_3_-terminal GST tag. Vectors were transformed into BL21 *E*. *coli* (Agilent, Santa Clara, CA), and peptide expression was induced for 2 hr with 1 mM IPTG upon reaching OD_600nm_ of 0.6 in a shaker flask grown at 30°C. Bacteria were lysed using Cell Lytic B (Sigma) in the presence of Complete Protease Inhibitors without EDTA (Roche). Peptide purification was performed using sequential nickel-NTA agarose and glutathione sepharose columns as previously described [15]. Four mL nickel NTA agarose was equilibrated with wash buffer (20 mM Tris pH 7.4, 600 mM NaCl, 40 mM imidazole, and 0.01% tween 20) before passing induced cell lysates over the column. The column was washed with 10 column volumes of buffer before eluting with TBS containing 250 mM imidazole. Samples were concentrated and buffer exchanged into TBS using 10 kDa cut-off filter Microcon centrifuge concentrators (EMD Millipore, Billerica, MA). Concentrated samples were then applied to a GST Spin-Trap column (GE Healthcare Life Sciences), washed with TBS, and eluted with 1 mM L-glutathione. Peptides were concentrated and buffered exchanged into TBS, and purity was analyzed by SDS-PAGE followed by staining with SYPRO RUBY (Invitrogen). Proteins were stored at -80°C until use.

### Peptide Kinetics

The rate of ADAMTS13 proteolysis of various mutant VWF73 peptides, at concentrations between 0.5 to 5 nM (at least 340-fold < K_M_), was determined using a discontinuous assay and AlphaLISA, as described above but with the following modifications. After stopping the reaction, 5 μl of 1/10 diluted reaction time points were added into wells of a 96-half well plate with 25 μl of a solution containing 10 μg/ml anti-HIS antibody-coated acceptor beads (Perkin-Elmer) and 0.4 nM biotinylated anti-GST antibody (Perkin-Elmer) in AlphaLISA buffer. The mixture was incubated for 1 hr at RT. Next, 20 μl of a solution containing 20 μg/ml streptavidin coated donor beads in AlphaLISA buffer was added to the wells before incubating for 30 min at RT. Plates were read as described above. Catalytic efficiency was calculated using Eq 1 and 2, as described above.

### In vivo Cleavage Analysis

Subcloning of FLAG and E tagged murine VWF (wtVWF) into pLIVE (Mirus Bio) has been previously described[27]. Mutations in wtVWF cDNA were generated with primers P7—P26, Table 1, and the QuikChange II XL site-directed mutagenesis kit (Agilent) according to the manufacturer’s instruction. Mutations were verified by sequencing using primer P3, Table 1. 129X1/SvJ mice or *Adamts13* ^-/-^ mice on a C57BL/6J genetic background (>20 generations) were hydrodynamically injected, and platelet poor plasma (PPP) was collected as previously described[27]. All animal procedures were approved by the University of Michigan’s Committee on Use and Care of Animals.

Except for *Adamts13* ^-/-^ mice, all hydrodynamic injections reported here used 129/Sv mice expressing full length ADAMTS13, unlike the commonly used C57 BL/6 strain that express a truncated and probably hypomorphic form of ADAMTS13[28, 29]. The phage library from which specific mutations were identified, contained human VWF cDNA sequence. However, due to the inability of human VWF to interact with murine platelets (through murine GPIb)[30] the mutations were introduced into murine VWF cDNA which is highly conserved with human VWF between p3’ and p11[27].

Plasma concentrations of FLAG-tagged VWF were quantitated by ELISA as previously described[27]. FLAG-tagged VWF in PPP was reduced (4% 2-mercaptoethanol) and subjected to SDS-PAGE on 6% tris-glycine gels (Invitrogen), transferred onto nitrocellulose (Bio-Rad), and analyzed by western blotting as previously described[27].

## Results

### Phage Library Screen

**Fig 1:**
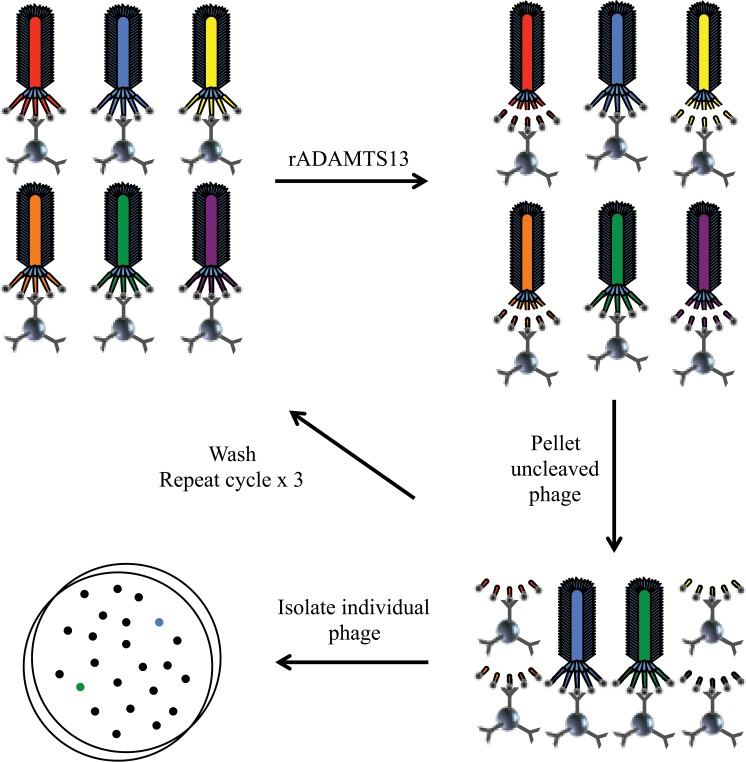
Phage screen process. Freshly prepared library phage where incubated with rADAMTS13 in cleavage buffer in the presence of anti-FLAG antibody coated beads. Uncleaved phage were separated from cleaved phage by centrifugation and washed. Phage on beads were exposed to three additional rounds of incubation with rADAMTS13. Individual phage were isolated by direct infection to K91 cells and plating on tetracycline containing NZY agar.

Three independent mutagenized VWF73 substrate phage display libraries were created and screened with rADAMTS13. The unscreened library depths were 5.0 x 10^5^, 1.0 x 10^5^ and 1.1 x 10^7^ colony forming units (CFU) while the average DNA mutation frequencies were 1.1, 2.6 and 2.5 mutations per VWF73 coding sequence (219 basepairs) respectively. Fig 1 illustrates the phage screening process and isolation of phage expressing VWF73 substrates resistant to ADAMTS13 cleavage. A total of 260 phage colonies from screening experiments were randomly selected for an ELISA-based secondary screen. Of the 260 phage clones screened by ELISA, 136 (52%) showed decreased relative cleavage compared to known wild-type VWF73 (wtVWF73) expressing phage controls. Of the 136 phage clones resistant to cleavage in the secondary screen, 122 (90%) contained unique sequences (S1 Fig). Phage bearing identical sequences were always derived from the same one of the three independent phage libraries consistent with an origin from a single “ancestral” phage. Six of 136 phage expressed wild-type VWF73 suggesting a false positive screening rate of 4%.

**Fig 2:**
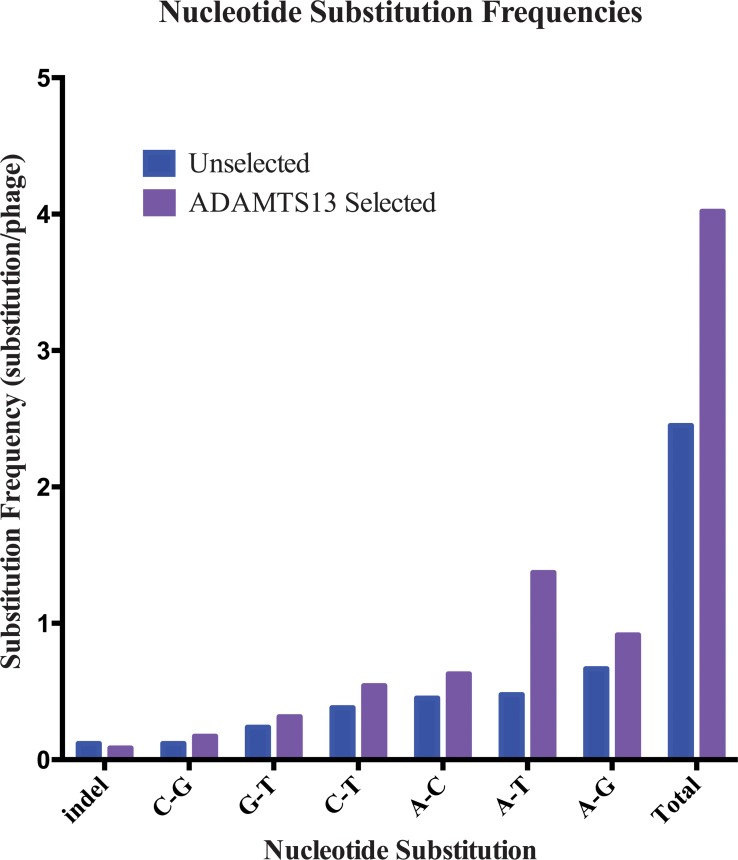
Mutation frequency in unselected and selected libraries. VWF73 encoding sequences (219 bp) from ADAMTS13 selected phage were compared to VWF73 encoding sequences from unselected phage derived from the same original library. There were 103 nucleotide changes in the 42 phage (2.45/phage) sequenced in the unscreened library compared to 141 mutations in 35 phage (4.03/phage) sequenced after selection by ADAMTS13. Sequences were aligned and mutations were binned into the substitution class using the MegAlign program. To obtain the number of substitutions per sequenced phage, the total number of mutations in each substitution class was divided by the number of phage sequenced in the unselected library (42) or the selected library (36).

Fig 2 compares the distribution of nucleotide substitutions in an unscreened and ADAMTS13 screened library. As expected, the screened library exhibited greater mutation frequency across all nucleotide substitution types, (p value = 0.0156, Wilcoxon matched pairs signed rank test), consistent with a selection for amino acid changes that cause resistance to proteolysis.

**Fig 3:**
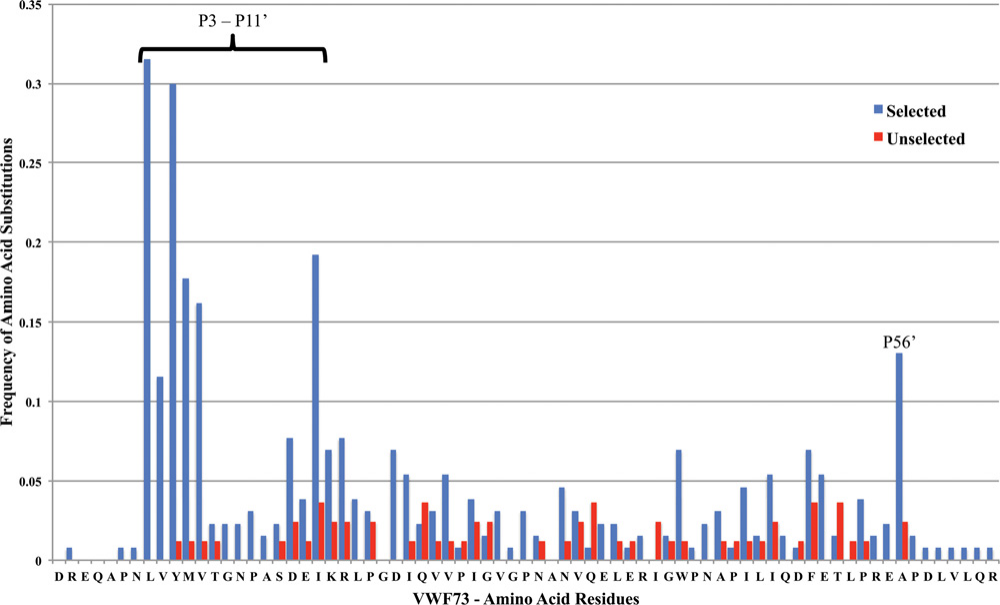
Mutation frequency across VWF73 phage. The mutation frequency was calculated at each position in 130 phage recovered from screening experiments for resistance to ADAMTS13 cleavage and in 82 phage randomly selected from unscreened libraries.

The ADAMTS13 selected phage expressed a total of 387 amino acid substitutions in the VWF73 sequence which resulted in an average of 3.2 amino acid substitutions per substrate. Fig 3 displays the mutation frequencies at each amino acid residue in phage selected for resistance to ADAMTS13 (selected) and in phage isolated from original libraries without ADAMTS13 selection (unselected). Although ADAMTS13 resistant phage had amino acid substitutions across the entire substrate, 163 (42%) amino acid substitutions occurred between the P3 and P11’ residues flanking the ADAMTS13 cleavage site (Fig 3 and Table 2). Among phage with substitutions between P3—P2’, only 21% had an additional substitution within the P3—P2’ while among phage with substitutions at the P11’ position, 72% expressed an additional substitution between P3 and P2’. Thirteen percent of selected phage had an amino acid substitution at P56’ and 65% of these phage also expressed one or more substitutions between P3 and P2’. This suggested that phage with mutations outside of the P3—P2’ sites generally required additional mutations within the P3—P2’ sites to be resistant to ADAMTS13 in the screen.

**Table 2:** Distribution of mutations in phage selected for resistance to ADAMTS13.

^a^ **Position (N)**	**Mutation**	**Count**
P3 (41)	L1603Q	24
	L1603R	9
	L1603M	2
	L1603P	2
	L1603V	2
	L1603W	1
	L1603del	1
P2 (15)	V1604D	9
	V1604F	2
	V1604G	1
	V1604I	1
	V1604L	1
	V1604S	1
P1 (38)	Y1605D	16
	Y1605N	12
	Y1605H	4
	Y1605P	2
	Y1605L	1
	Y1605R	1
	Y1605S	1
	Y1605T	1
P1’ (23)	M1606K	10
	M1606T	8
	M1606V	3
	M1606W	1
	M1606P	1
P2’ (21)	V1607D	17
	V1607A	3
	V1607S	1
P11’ (25)	I1616N	14
	I1616T	6
	I1616F	2
	I1616V	2
	I1616S	1
P56’ (17)	1661D	17

^a^ Position of amino acid residue is relative to ADAMTS13 cleavage site.

**N**: Number of sequenced phage with mutations at this residue

**Count**: number of sequenced phage with specific amino acid substitution.

### Quantification of select VWF73 variants

**Fig 4:**
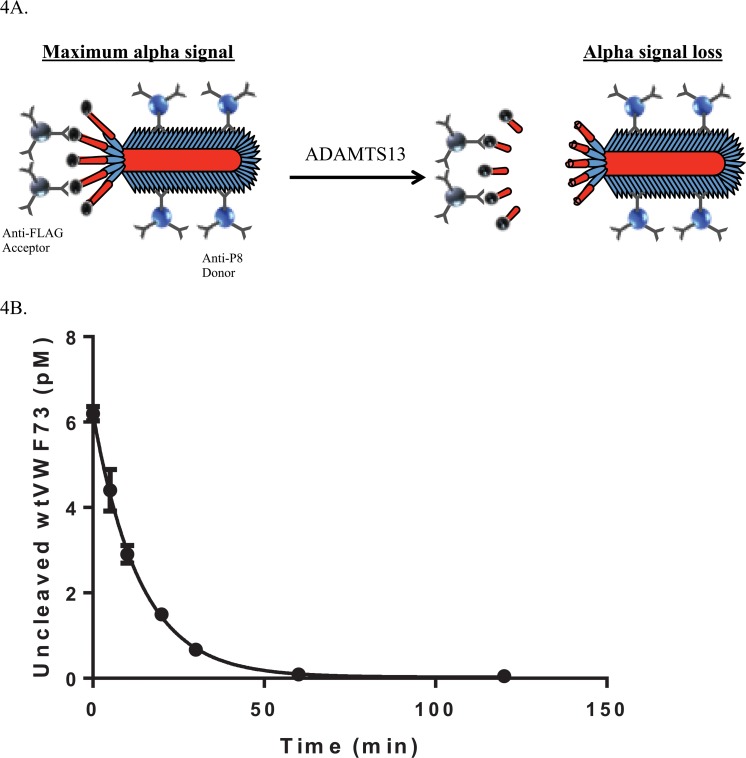
Reaction time course of ADAMTS13 and wtVWF73 M13 phage. *A*, Schematic representation of AlphaLISA reaction in mutant VWF73 bearing phage. Cleavage of the displayed VWF73 peptide was determined by a loss of Alpha signal caused by the release of the FLAG tagged NH_2_-terminal VWF73 fragment and subsequent separation of donor and acceptor beads. *B*, M13 phage displaying wtVWF73 (6 pM) were reacted with ADAMTS13 (2 nM) in ADAMTS13 reaction buffer B at 37C. Aliquots were removed into stop buffer containing EDTA at indicated time points. Data were fit by a first-order non-linear regression model and plotted in the Prism software package.

The VWF73 variants cloned into fUSE55 phage should be expressed as fusions to the PIII protein and “displayed” in 3–5 copies per phage particle, Fig 1. Despite this stoichiometry, cleavage of wild-type VWF73-expressing phage demonstrated classic substrate consumption kinetics when exposed to ADAMTS13, as detected by AlphaLISA, Fig 4.

Because phage-displayed peptide proteolysis obeys predicted substrate behavior, we compared the catalytic efficiencies of wild type VWF73 phage to selected mutant VWF73 phage clones recovered from our ADAMTS13 cleavage screen, S1 Fig. The catalytic efficiency obtained for wild type VWF73-displaying phage was 1.5 x 10^7^ M^-1^min^-1^, comparable to published values for recombinant peptide [17]. Six of the eight mutants studied (clones #15, #33, #40, #64, #69 and #81, S1 Fig) exhibited no detectable proteolysis, despite incubation with 100 nM ADATMS13 for 600 min, Table 3. VWF73 phage with the mutations M1606T and I1616N (Clones #90 and #111) had measurable rates of cleavage when exposed to 100 nM ADAMTS13, leading to catalytic efficiencies that are 50- to 100-fold lower compared to wtVWF73, Table 3. Phage expressing R1597W, a common mutation identified in VWD Type 2A group 2 patients, were generated by PCR and demonstrated a similar catalytic efficiency to wtVWF73.

**Table 3:** Phage and VWF73 peptide enzyme kinetics K_cat_/K_M_ (M^-1^min^-1^).

**VWF Variants**	**Phage (** ^c^ **Clone #)**	^d^ **Screen Derived Peptide**	**Peptide**
**Wildtype**	^a^1.5 x 10^7^ ± 6.2 x 10^6^	^a^1.4 x 10^7^ ± 7.7 x 10^6^	
R1597W*	^a^2.4 x 10^7^ ± 5.9 x 10^6^	NA	^a^3.5 x 10^7^ ± 1.6 x 10^7^
**L1603Q**	^b^Not Detected (15)	^b^Not Detected	^a^2.9 x 10^5^ ± 3.1 x 10^5^
**L1603R**	^b^Not Detected (40)	^b^Not Detected	^a^5.6 x 10^4^ ± 9.4 x 10^3^
**Y1605D**	^b^Not Detected (64)	^b^Not Detected	^a^1.2 x 10^5^ ± 9.9 x 10^4^
**M1606T**	^a^7.4 x 10^5^ ± 2.2 x 10^5^ (90)	^a^1.5 x 10^5^ ± 1.0 x 10^5^	NT
**I1616N**	^a^6.3 x 10^5^ ± 2.3 x 10^5^ (111)	^a^4.1 x 10^5^ ± 2.6 x 10^5^	^a^2.9 x 10^5^ ± 1.1 x 10^5^
**L1603R/Y1605N**	^b^Not Detected (33)	^b^Not Detected	^b^Not Detected
**Y1605N/M1606T**	^b^Not Detected (69)	^b^Not Detected	^b^Not Detected
**Y1605R/M1606T**	^b^Not Detected (81)	^b^Not Detected	^b^Not Detected

^a^ denotes reaction conducted with 10 nM ADAMTS13.

^b^ denotes reaction conducted with 100 nM ADAMTS13.

^c^ Clone #, from S1 Fig.

^d^ Peptide sequence derived from phage screen. Differs from Peptide due to the presence of additional mutations outside P3-P2’, see S1 Fig.

* Clone created by site—directed mutagenesis. Not derived from screen.

NA, Not applicable

NT, Not tested

In order to determine if the kinetics measured using phage particles as substrates were comparable to peptide substrates containing the same amino acid substitutions, analyses of purified peptide substrates were performed. The results obtained with peptide substrates were consistent with the data using corresponding phage substrates. The wtVWF73 peptide exhibited a k_cat_/K_M_ value of 1.4 x 10^7^ M^-1^min^-1^, similar to the value obtained as a phage substrate (1.5 x 10^7^ M^-1^min^-1^, Table 3). Similarly, R1597W, was indistinguishable from wtVWF73 as a phage substrate and as a peptide substrate. Peptide or phage substrates with mutations that include M1606T and I1616N exhibited 50- to 100-fold reduction in k_cat_/K_M_ compared to wtVWF73. The six mutants with no detectable cleavage in phage also had no detectable cleavage as peptides.

The VWF73 variants isolated from the phage screen contained multiple amino acid substitutions that may influence the rate of proteolysis by ADAMTS13. Next, we focused our analysis on those amino acid changes only within P3—P2’. Unlike the screen derived phage or corresponding peptides with multiple substitutions, peptides expressing only L1603Q, L1603R or Y1605D substitutions demonstrated detectable cleavage with catalytic efficiencies 100 to 500-fold less than wtVWF73, Table 3. Similar to screen derived phage or peptides with two mutations in the P3—P2’, clone #33, #69 and #81, S1 Fig, there was no evidence of proteolysis of the corresponding peptides with only Y1605N/M1606T, Y1605R/M1606T, or L1603R/Y1605N substitutions despite incubation with 100 nM ADAMTS13 for 600 minutes. These results suggest interactions between single amino acid substitutions within P3—P2’ and other substitutions outside of this subsite.

### Cleavage of full-length mutant VWF substrates in vivo

**Fig 5:**
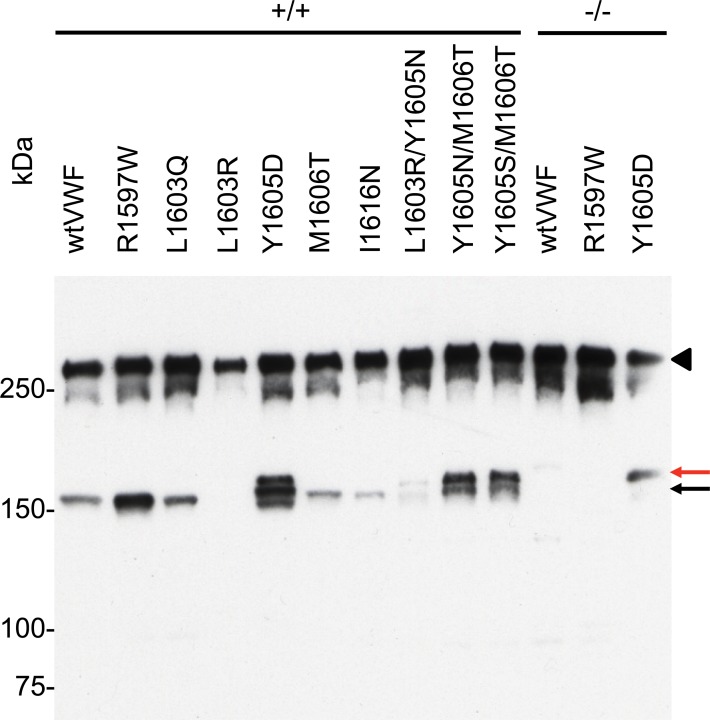
Mutations in VWF73 conferring ADAMTS13 resistance fail to prevent proteolysis of multimeric VWF in mice. Equal mass (10 ng) of VWF in PPP collected 1 week after hydrodynamic injection were evaluated for ADAMTS13-dependent proteolysis. Plasma concentrations of hepatically-derived VWF ranged from 5.4 to 21.7 μg/mL for the samples shown. Uncleaved (black arrowhead), ADAMTS13-dependent (black arrow), and ADAMTS13-inedpendent proteolytic products (red arrow) were observed. *Adamts13+/+* mice were 129X1/SvJ, and *Adamts13-/-* mice were maintained on a C57BL/6J genetic background.

**Fig 6:**
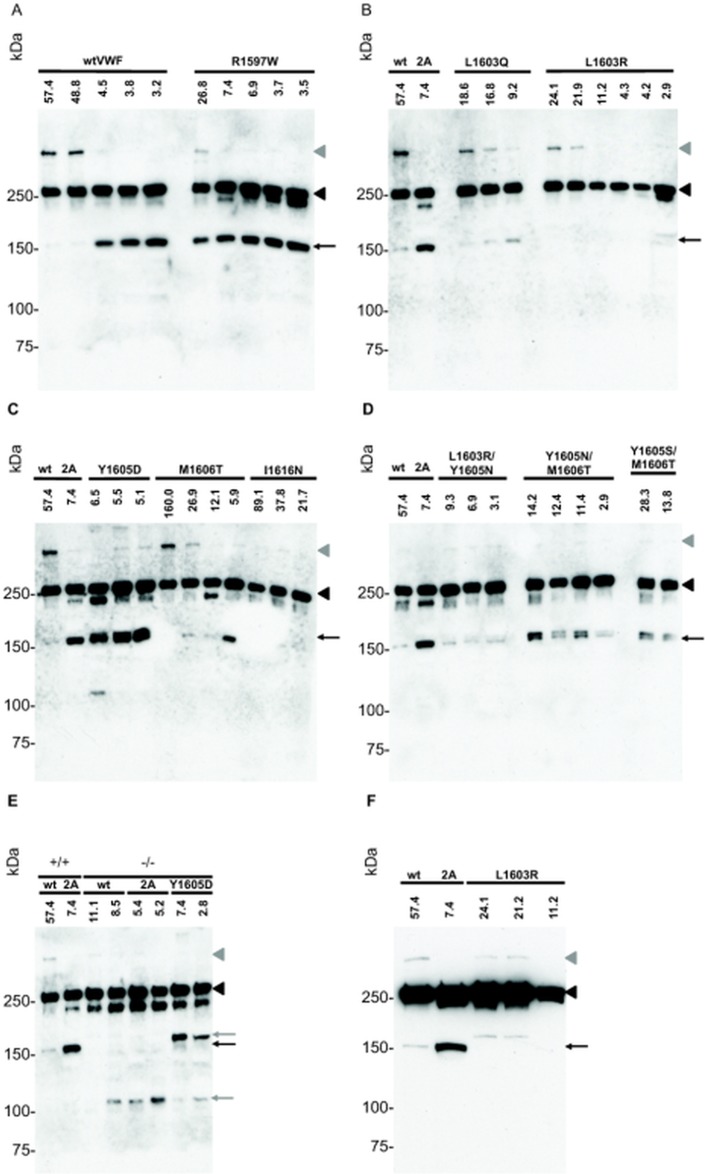
*In vivo* proteolysis of VWF mutants. FLAG tagged, plasma VWF, *A-E*; 10ng load, F; 25ng load, were examined for proteolysis in 129X1/Sv *Adamts13 +/+* or *Adamts13*-/- mice by reducing SDS-PAGE and western blotting. Each lane represents an individual mouse with the VWF mutation(s) and VWF concentration in PPP as measured by ELISA (ug/mL). 10 ng of VWF was loaded per lane. Several forms of VWF were observed: proVWF (i.e., propeptide bound; gray arrowhead), uncleaved mature VWF (black arrowhead), Adamts13-mediated proteolysis (black arrow), and Adamts13-independent proteolysis (gray arrow). In the 2 left most lanes of *B*-*F*, wtVWF and R1597W are the same as the 1^st^ wtVWF and 2^nd^ R1597W of *A*, respectively.

We further examined the effects of selected amino acid substitutions on circulating, multimeric VWF which, unlike VWF73, contains the complete A2 domain and extended exosites for ADAMTS13 [31]. Hydrodynamic injection of plasmids encoding single or double P3—P11’ site substitutions resulted in levels of expression between 2.8 μg/mL and 160 μg/mL in individual mice. Western blot of plasma from injected mice under reducing conditions demonstrated C-terminal ADAMTS13-mediated cleavage products at the expected 176 kDa size from wtVWF and the type 2A VWD (R1597W) mutant substrate (Figs 5 and 6A and 6E). As expected there was an apparent increased cleavage of R1597W compared to wtVWF. When wtVWF or R1597W were expressed in *Adamts13* ^-/-^ mice, no 176kD bands were observed, confirming the derivation of this C-terminal cleavage product from ADAMTS13 mediated cleavage, Fig 5. Mice expressing L1603R exhibited no detectable 176 kDa cleavage product. However, a slightly larger (190 kDa) product was observed in three of six mice injected with L1603R encoding plasmids, Fig 6B and 6F. In contrast to the *in vitro* cleavage of mutant VWF73 peptides, *in vivo* proteolysis of multimeric VWF carrying L1603Q, M1606T, and I1616N substitutions appeared comparable to wtVWF, Figs 5 and 6B and 6C and 6D. Additionally, alternate cleavage patterns were observed with Y1605D, which demonstrated a higher molecular weight (190 kDa) cleavage product in all tested mice. These same proteolytic products were observed for Y1605D expressed in *Adamts13* ^-/-^ mice, demonstrating their derivation through the activity of another protease independent of ADAMTS13, Figs 5 and 6E.

## Discussion

Although previous investigations into ADAMTS13 substrate specificity have analyzed a limited set of amino acid substitutions within the VWF A2 domain, this is the first report of an unbiased screen of ADAMTS13 specificity in a large library of VWF mutations. Although ADAMTS13 has increased activity at lower pH *in vitro*[32], all experiments in this report were conducted at pH 7.4 to better mimic the pH in circulating blood. The results of our screen demonstrated the importance of residues outside of but close to the P1-P1’ sites (P3, P11’) for ADAMTS13 substrate recognition. The additive depth of the libraries suggested that every possible amino acid substitution within VWF73 was represented. This approach is limited by the lack of mammalian post-translational modification including glycosylation and the inability to screen large proteins. However, phage expressing wtVWF73 demonstrated kinetics comparable to purified VWF73 peptides. Surprisingly, the valency of the phage-expressed substrates, which is predicted to be between 3–5 copies per phage, did not seem to affect the first order rate constant for wtVWF73. This is likely due to the excess of ADAMTS13 used in the reactions. We hypothesized that mutations between P3—P11’ dominated the selective pressure in our screen and we focused our subsequent kinetic and *in vivo* cleavage experiments on selected clones with amino acid substitutions in between P3—P11’.

The P3 residue (L1603) was the most frequently mutated site in the set of resistant phage recovered from the three independent library screens. The L1603R mutant showed the greatest reduction in k_cat_/K_M_ (178-fold) compared to all other tested single mutations and was the only mutant substrate demonstrating a clear resistance to cleavage in the murine circulation as full-length VWF. Previous studies have demonstrated that the substitution of leucine for serine, alanine, asparagine or lysine at 1603 reduces or abolishes cleavage of a 115 amino-acid fragment of VWF [31]. Our experiments analyzing L1603R and L1603Q confirm the importance of this position and suggest that arginine substitutions strongly inhibit cleavage, both in the context of VWF73 and in full-length multimeric VWF.

R1597W is the most frequently reported mutation in patients with VWD Type 2A. This disorder is characterized by a loss of the largest molecular weight VWF multimers in circulation. Though the *in vivo* experiments confirmed the increased susceptibility of full-length R1597W VWF to ADAMTS13, the cleavage of R1597 was very similar to WT VWF in phage and VWF73 peptides. Taken together, these data suggests that, although the R1597W mutation resides in VWF73 at residue P9 near the cleavage site, it may cause a conformational change improving substrate specificity only in the context of a larger A2 domain fragment or multimeric VWF. This finding is consistent with a previous report demonstrating A2 domain conformational changes caused by R1597W [33].

We recovered 17 phage (13% of sequenced phage) with the V1607D mutation. In humans, this VWF mutation is associated with VWD Type 2A group 1. In contrast to VWD 2A group 2 mutations such as R1597W, group 1 mutations result in a misfolded protein, retained in the ER, thus interfering with VWF multimer assembly and secretion [34]. Resistance of V1607D-bearing phage to ADAMTS13 could be the result of a conformational change due to the hydrophopic to acidic amino acid substitution near the scissile bond.

Crystallization of a truncated form of ADAMTS13 and modeling of ADAMTS13 VWF interactions predicted three exosites in ADAMTS13 [14, 35]. The proposed exosite-1 in this model would interact with the most sensitive cluster in VWF between the P3 and P11’. Interestingly, we did not identify a significant increase in the frequency of mutations in the area of VWF73 that was predicted to interact with exosite-2. This may be due to the specific conformations in exosite-2 in our phage screen or a limited power to detect mutations in this region with small effect sizes.

Amino acid substitution at the P56 residue, A1661D, was observed in all three independent library screens. The P56 residue was modeled to interact with a proposed exosite in the spacer domain of ADAMTS13 [16]. The A1661D mutation introduces an acidic amino acid into the predicted alpha helix formed in this region of VWF73, which may reduce its interaction with the hydrophobic exosite core. No other mutations were identified in the screen at this C-terminal subsite, which may have been due to limited library diversity at this site or the influence of the nearby phage PIII protein residues.

The results from the *in vivo* experiments suggest important differences in specificity between phage/peptide VWF73 and full-length multimeric VWF substrates. In addition to the discrepant cleavage patterns observed for R1597W variants described above, we demonstrated that several variants with no evidence of cleavage in phage or peptide have detectable cleavage products in the context of multimeric VWF in flowing blood. The VWF73 substrate may omit important subsites in the A2 domain (N-terminal and C-terminal) that interact with ADAMTS13 exosites and play a role in determining substrate specificity *in vivo*. In addition to other exosite interactions, the P3 to P11’ mutations that were studied here may have destabilized the A2 domain and changed the dynamic unfolding in circulation that is required for ADAMTS13 cleavage [33, 36]. Additionally, the hydrodynamic tail vein injection process may not produce the same distribution of molecular weight multimers that are normally produced by the endothelium. Since ULVWF is thought to be more susceptible to cleavage by ADAMTS13 [37], a decreased amount of ULVWF in hydrodynamically expressed VWF could have led to a decreased percentage of cleavage detected in these experiments. Taken together, our findings in selected full-length mutant substrates *in vivo* suggest an important role for other exosite interactions outside of the minimal substrate VWF73 and may have implications when using VWF73 as a substrate to measure ADAMTS13 activity clinically.

Although mutations within the *VWF* gene resulting in reduced susceptibility to ADAMTS13 cleavage have not been previously reported in patients with TTP, our results suggest that such mutations could explain a subset of patients with a TTP-like syndrome but normal plasma ADAMTS13 activity. This hypothesis could be tested in the future through appropriate gene editing experiments in the murine *VWF* gene.

This report focused on the discovery of VWF residues that reduce proteolysis by ADAMTS13. However, this phage system could also be used to identify VWF73 fragments more sensitive to ADAMTS13, which could facilitate the development of improved ADAMTS13 activity assays. Additionally, modification of the phage library to include larger VWF fragments might uncover other subsites in VWF that contribute to substrate specificity. Finally, this phage display system could be used to map important substrate residues for a wide variety of other biologically relevant proteases.

## Supporting Information

S1 Fig Comparison of VWF73 sequence from screen derived phage to wild type. Individual phage colonies that were resistant to ADAMTS13 were Sanger sequenced. DNA sequence was translated into peptides using the EditSeq software package. Phage protein sequences were imported into the MegAlign software package and aligned with wtVWF73 protein sequence. Phage are ordered by the location of the first missense mutation. Only amino acids differing from the wtVWF73 are noted with letters.
(EPS)
